# Home-based exercise program and Health education in patients with patellofemoral pain: a randomized controlled trial

**DOI:** 10.1186/s12891-023-07027-z

**Published:** 2023-11-18

**Authors:** Qiao-Mei Hong, Hao-Nan Wang, Xi-Hui Liu, Wen-Qi Zhou, Xiao Zhang, Xiao-Bing Luo

**Affiliations:** 1 Department of Sport Medicine, Sichuan Province Orthopedic Hospital, Chengdu, Sichuan Province China; 2 Faculty of Sport Medicine and Rehabilitation, Beijing Sport University, Beijing, China; 3 Faculty of Kinesiology, University of Calgary, Calgary, AB Canada

**Keywords:** Patellofemoral pain, Anterior knee pain, Home-based exercise, Health education, Rehabilitation

## Abstract

**Background:**

Patellofemoral pain (PFP) is one of the most common disorders of the knee joint. Home-based exercise is an effective intervention to achieve self-management for chronic diseases. This study evaluated the effects of home-based exercise and health education in patients with PFP.

**Methods:**

Patients who had PFP were randomly allocated to an intervention group (IG) or control group (CG). Patients in the IG received a 6-week tailored home-based exercise program with health education via remote support, while patients in the CG group only received health education. Clinical outcomes were compared using the Anterior Knee Pain Scale (AKPS) to measure function and the Visual Analog Scale (VAS) to measure “worst pain” and “pain with daily activity”. Muscle strength was measured according to the peak torque of the knee muscles using an isokinetic system.

**Results:**

Among a total of 112 participants screened for eligibility, 38 were randomized and analyzed, including 19 participants in the intervention group and 19 participants in the control group. There were no significant differences in baseline characteristics between the groups. At 6-week follow-up, the intervention group showed a greater worst pain reduction (between-group difference, -19.3 [95%CI, -23.2 to -15.5]; *P* < 0.01) and pain with daily activity (between-group difference, -22.9 [95%CI, -28.3 to -17.4]; *P* < 0.01) than the control group. Similarly, the intervention group had better improvements in AKPS (between-group difference, 9.0 [95%CI, 4.1 to 13.9]; *P* < 0.01) and knee extensor strength (between-group difference, 20.1 [95%CI, 14.5 to 25.8]; *P* < 0.01), compared to the control group. No adverse events were reported.

**Conclusion:**

Home-based exercise and health education resulted in less pain, better function, and higher knee muscle strength compared with no exercise in patients with PFP. A large randomized controlled trial with long-term follow-up is required to confirm these findings.

**Trial registration:**

Chinese Clinical Trial Registry, ChiCTR2200056224 (https://www.chictr.org.cn/showproj.aspx?proj=135506). Registered on February 1, 2022.

## Background

Patellofemoral pain (PFP) is a common musculoskeletal disorder of the knee. PFP is characterized by diffuse retropatellar and peripatellar pain resulting from physical and biomechanical changes altering the stress and loading of the patellofemoral joint [1]. The symptom is aggravated by activities that increase stress and loading on the patellofemoral joint, such as stair climbing, squatting, running, and prolonged sitting with bent knees [2]. The annual prevalence of PFP has been reported to be 22.7% in the general population, and 28.9% in adolescents [3]. This condition affects many aspects of daily life in both the athletic and nonathletic populations [4]. Although the etiology of PFP remains unclear, it is linked to patellar maltracking and increased patellofemoral stress [5], which are caused by abnormal muscle morphology [6, 7], muscular performance [8, 9] and altered biomechanical factors [10–12].

Growing evidence showed that exercise is considered an effective intervention to treat patients with PFP [13]. Exercise therapy targeting the hip and knee is recommended in the management of PFP to restore muscle balance [1]. Both supervised hip- and knee-targeted exercise programs are beneficial and provide short-term pain relief and function improvements for PFP patients [14, 15]. In addition, a combined hip and knee exercise program seemed to be effective and superior to knee strengthening alone for decreasing pain and improving activity in individuals with PFP [16]. Hip and quadriceps exercises have a better effect if the exercises are performed more frequently [17]. Home-based exercise programs are an effective and low-cost approach to managing patients that have been applied successfully for a variety of conditions, including stroke [18], cardiovascular diseases [19], risk of falling [20], osteoarthritis [21]. Particularly during the COVID-19 pandemic, home-based programs have gained interest, importance, and engagement among patients [22]. Thus, a home-based exercise program may be beneficial to assist self-management for PFP patients. A previous study found that arthroscopy used in combination with a home exercise program was no better than when the home exercise program was used alone to treat chronic PFP [23]. Recently, Kölle et al. [24] demonstrated that PFP patients experience pain relief, improved function, and reduced imbalance of delayed onset or reduced activity of the quadriceps after 12 weeks of home-based online treatment. To date, the available evidence has been limited to investigating a tailored home-based exercise program in patients with PFP.

Therefore, the aim of this study is to evaluate the effectiveness of a six-week home-based program on the pain and function, as well as isokinetic muscle strength of the knee joint in patients with PFP. Our hypotheses were that a home-based exercise program would relieve patients’ pain, and improve their self-reported function AKPS and muscle strength, compared to a health education intervention.

## Methods

### Study design

This study had a randomized controlled design and was conducted between March and October 2022 at the Department of Sport Medicine of Sichuan Province Orthopedic Hospital. The Sichuan Province Orthopedic Hospital is a prominent non-profit hospital located in Sichuan Province, China. It holds the distinction of being the Approved Hospital for supporting National Team Athletes designated by the Chinese Olympic Committee. All participants provided written informed consent proved by Department of Sport Medicine of Sichuan Province Orthopedic Hospital before the trial, and the rights of the participants were protected. Outcome measurements were performed at baseline and at the end of the six-week treatment. All participants were allocated to either (1) a group that completed home-based exercise with health education (Intervention group) or (2) a group that included only patient health education but no exercise (Control group). Participants in the intervention group attended 18 exercise sessions (3 sessions/week) for 6 weeks. This study was approved by the Ethics Committee of Sichuan Province Orthopedic Hospital (Ethics Approval No. KY2021-031-01) and registered at the Chinese Clinical Trial Registry (ChiCTR2200056224, 01/02/2022).

### Consort statement and flow chart

**Fig. 1:**
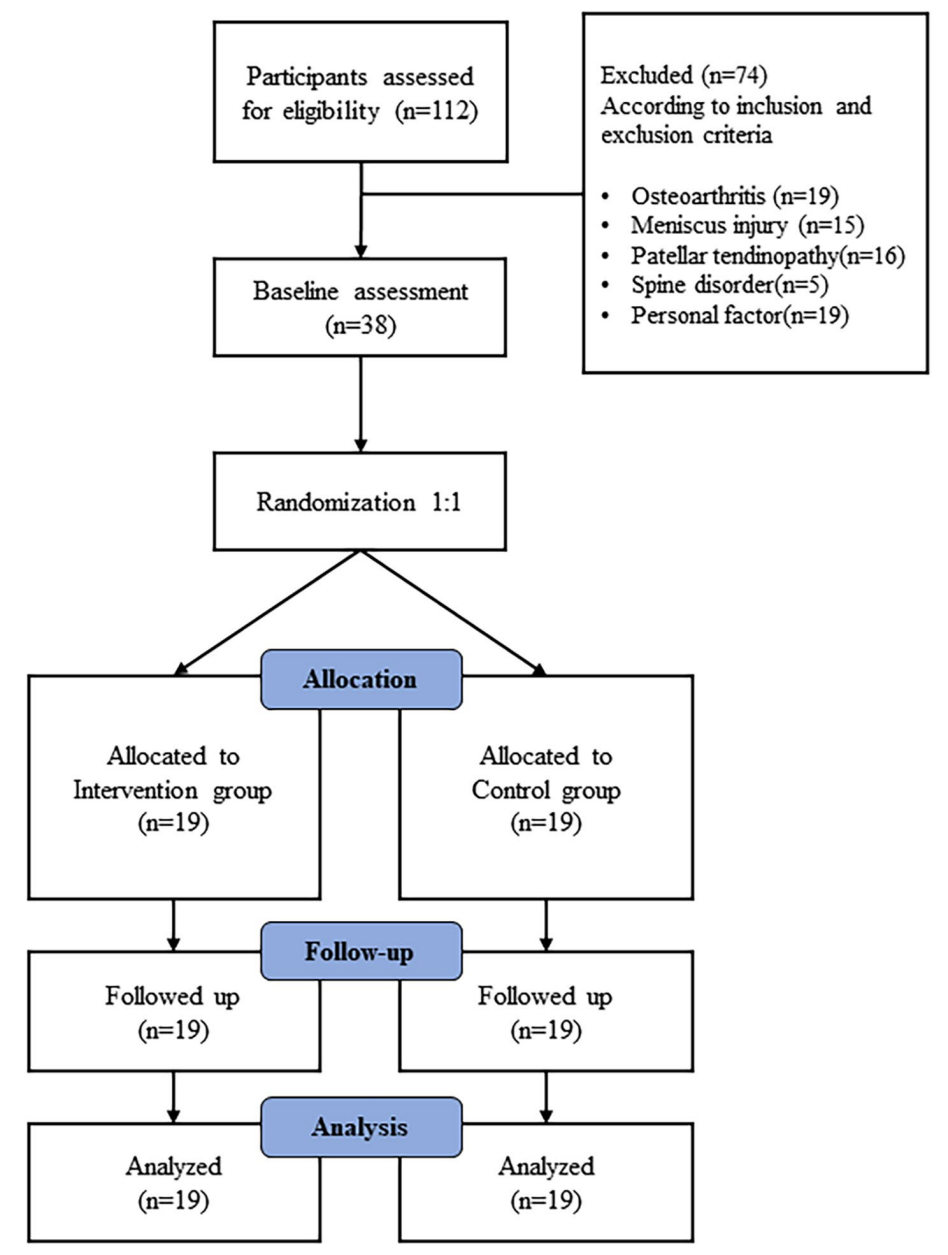
Flowchart of the study design

This study was designed and reported in line with the CONsolidated Standards of Reporting Trials (CONSORT) guideline and CONSORT PRO extension recommendations for reporting randomized trials (Fig. 1).

### Participant enrollment

Initial recruitment involved 112 potential patients with a diagnosis of unilateral or bilateral PFP from the Department of Sport Medicine of Sichuan Province Orthopedic Hospital. PFP was diagnosed by an experienced physician. For participants with bilateral PFP, the affected side with worse pain intensity (assessed using the Visual Analogue Scale) was identified as the affected leg. Potential participants were then referred by clinicians and screened by a senior physiotherapist according to the inclusion and exclusion criteria. Finally, 38 male and female patients with PFP were recruited for this study.

#### Inclusion criteria

The inclusion criteria were as follows [25, 26]: (1) 18–40 years of age; (2) unilateral or bilateral anterior knee pain over four weeks with a minimum pain level of 3/10 on a visual analogue scale (VAS); (3) insidious onset of symptoms unrelated to a traumatic accident; (4) presence of pain in any two activities of running, jumping, squatting, kneeling, walking upstairs/downstairs or prolonged sitting; and (5) the presence of 2 or more symptoms during following physical examinations: pain during apprehension test, pain during patellar compression test, crepitation during the compression test, tenderness upon palpation of the posterior surface of the patella or surrounding structures, or pain during resisted knee extension.

#### Exclusion criteria

The exclusion criteria were as follows [27]: (1) history of patellar dislocation, subluxation, chondral damage, ligament laxity or other knee joint injuries; (2) suspicion of patellar tendinopathy, localized pain on patellar tendon and relieved pain during knee resisted extension; (3) previous knee surgery or arthritis; (4) any other invasive procedure in the affected knee, including arthroscopy or intra-articular injection in the past 12 months; (5) history of physical therapy or a physical strengthening procedure of the affected knee in the past six months; (6) received nonsteroidal anti-inflammatory drugs in the past three months; (7) any neurological, heart, or vascular disease, such as blood coagulation disorders; (8) other acute or chronic disorders or psychiatric conditions that will affect physical or cognitive functions; or (9) pregnancy.

### Randomization and blinding

Participants were randomly allocated to one of the two study groups at a ratio of 1:1. Randomization was performed by a person independent of the study. The number sequence was generated using SAS software (SAS Institute, Cary, NC, USA) and placed in a sequentially numbered, sealed, opaque envelope prior to the recruitment of participants. Once the participants completed the informed consent process, demographic information, and baseline measures, envelopes were opened by one member of the research team not involved in the processes of measurement and intervention.

Due to the nature of the interventions in this study, the participants and therapists involved in home exercise and health education was not blinded. Participants presented to the hospital for baseline and six-week assessments conducted by a physiotherapist who was blinded to the treatment allocation. In addition, participants were informed not to discuss their intervention content at any time during the study. Unblinding was allowed in the case of severe adverse events and was reported as part of the results of this study.

## Outcome measures

### Primary outcome measure

#### Pain

Pain was measured by asking participants to draw a line on a 100 mm VAS to represent their worst pain during the previous week and pain with activities of daily living (ADL) (e.g., squatting, stair descent, and after 30 min of sitting) [28]. Test-retest reliability intraclass correlation coefficients (ICCs) of VAS worst pain and pain with activity were 0.76 and 0.83, respectively [29].

### Secondary outcome measure

#### AKPS

The knee self-report function was measured using the Anterior Knee Pain Scale (AKPS) [30]. This is a 13-item self-report questionnaire that assesses mobility capacity across six activities and related symptoms of PFP. The maximum score of the AKPS is 100, and a lower score indicates worse symptoms and functions of the knee. The test-retest reliability ICCs of this measure is 0.81 [29].

#### Muscle strength

Knee muscle strength was evaluated by maximal contractions using an isokinetic test system (IsoMed 2000, D&R Ferstl GmbH, Hemau, Germany). Before the test, the participants will perform three submaximal repetitions for familiarization. During the test, they were asked to perform five constant flexion and extension motions at their maximum effort using a concentric-concentric contractions model without gravity compensation at angular velocities of 60°/second. Knee muscle strength was evaluated by peak torque normalized to individual body mass (Nm/kg ×100). The test-retest reliability ICCs of the isokinetic knee muscle strength assessment was 0.94 [31].

#### Adherence

Exercise adherence was reported descriptively as a percentage of the total number of prescribed intervention sessions completed [32]. Participants recorded each of the scheduled sessions that they attended and filmed the video-based exercises log. At the end of this trial, after the physiotherapists checked the videos, the percentage of exercise sessions attended was calculated. The adherence to health education is documented by the therapist after each remote support session based on the patient’s level of engagement, the percentage of health sessions attended was calculated.

### Interventions

#### Intervention group

Participants in the intervention group completed one-on-one sessions with a physiotherapist to learn the technique of each exercise and 18 exercise sessions over 6 weeks, with 3 sessions per week (Table 1). In addition to face-to-face instruction, the participants were provided with a video with instructions for each exercise. For the duration of six weeks, the participants were asked to continue their previous activities without aggravating their knee symptoms. Patients in the study were permitted to use temporary pain-killer and/or anti-inflammatory medication as necessary. However, they were asked to abstain from taking any medication for 72 h prior to outcome measurements were conducted. During the period of this study, all participants were prohibited from engaging in any other co-intervention (including herbal medicine, Traditional Chinese medicine, and Traditional Chinese pharmacology).

**Table 1:** Exercise program performed by the intervention group

Hamstrings stretching, 3 repetitions of 30 s
Bridge with isometric contraction of the transversus abdominis-CORE training, 3 sets of 60 s
Sensori-motor training (standing) on cushion, 3 sets of 30 s
Hip abduction with weights (side lying), 3 sets of 15 repetitions+
Calm exercises (side lying) with an elastic band, 3 sets of 15 repetitions+
Calf raises with weights (standing), 3 sets of 15 repetitions+
VMO selected knee extension, 3 sets of 15 repetitions+
Seated knee extension with an elastic band, 90°–45° of knee flexion, 3 sets of 15 repetitions+
Single leg squat against wall, 0°–60°, 3 sets of 60 s+

+The load was adjusted every 2 weeks to maintain an effort of perception between 6 and 7 on the OMNI scale

Since knee strength is associated with PFP, most exercises in this program are strength-based exercises that included strength, stretching, and core stability exercises. Before the exercise session, the participants performed a warm-up by cycling or walking for 10 min to prevent injuries before the formal exercise protocol. To minimize patellofemoral joint stress while performing quadriceps exercises, a single leg squat against the wall was performed to between 0° and 60° of knee flexion, and leg extension was performed to between 90° and 45° of knee flexion [33]. In addition to quadriceps exercises, distal joint, proximal joint, and core muscle strength training were performed. Current evidence has shown that when biomechanical changes occur in patients with PFP [11], combining hip exercises with knee exercises is more effective than knee exercise alone [16]. Because low flexibility is one of the risk factors of PFP, stretching exercise training was adopted in this study. To provide a tailored exercise program, the program was adjusted every two weeks through one-to-one guidance by a physiotherapist. The intensity of each exercise was assessed using the OMNI scale and maintained an effort of perception between 6 and 7 (Table 1). The OMNI scale is indeed a tool used to assess and control intensity via monitoring the perceived exertion during exercises. The test-retest reliability of the OMNI scale for elastic bands exercise was 0.72–0.78 [34, 35]. Moreover, the pain intensity of each exercise was monitored using VAS during the exercise, with some pain considered acceptable [27]. When pain exceeds 20 mm on a 100 mm scale, the time and repetitions for each group are reduced until the OMNI scale rating decreases by one point.

Physiotherapists remotely reviewed the participants progress on intervention, adjusted the load of exercises if necessary and provided health education via 3 biweekly health education sessions. Health education session was delivered by WeChat APP (Tencent Computer System Co. Ltd., Shenzhen, China) and booklets that covered the information of clinical manifestations, risk factors, treatment of PFP, and benefits of exercise on PFP.

#### Control group

The individuals in the control group attended similar 3 biweekly health education sessions related to protecting their knee joints during activities in daily life via WeChat APP for six weeks. The sessions introduced the basic concept of PFP and the methods used to manage the risks of PFP, but their health education materials did not include exercise information or exercise instructions.

### Sample size

The sample size was estimated prior to initiating the trial by comparing the differences between two independent means using G-Power software (version 3.1.9.6, Heinrich-Heine-Universität Düsseldorf, Düsseldorf, Germany). The predetermined difference between the two groups was 20 mm of 100 mm VAS in pain intensity [29], which is above the minimal clinically important difference in patients with PFP. The parameters utilized to calculate the sample size included a standard deviation of 20 mm [36], a type I error of 5% (α = 0.05), and a power of 80% (β = 0.8), and the allocation ratio is 1:1. Accordingly, the minimum sample size of this study was set to 17 participants per group, the noncentrality parameter δ is 2.92 and the critical t is 2.04. With a possible withdrawal rate of 10%, the final sample size was set to 19 patients per group.

### Statistical analysis

The data were analyzed with SPSS 26.0 (SPSS Inc., Chicago, USA). The intention-to-treat principle was not adopted because no participants dropped out during the trial. The comparison between groups at the baseline was performed by using independent sample *t* tests for continuous variables and chi-square tests for categorical data (e.g., gender). All continuous variables followed a normal distribution using the Shapiro–Wilk test. For between-group comparisons, we utilized independent samples t-tests to compare the changes from PRE to POST tests (delta scores) within each group in pain, AKPS, and muscle strength. For within-group comparisons, we conducted paired t-tests to compare the continuous variables PRE and POST interventions. Data are presented as mean (SD), except when stated otherwise. The level of significance is set at *P* values < 0.05 for all data.

## Results

The descriptive data of the participants at the baseline were shown in Table 2. Of the 112 patients were recruited but ineligible or did not wish to participate, and 38 patients were enrolled. Figure 1 showed a flowchart of numbers of patients at different points in the study and the reason of patients excluded in the study. There was no significant difference between the groups at baseline for gender, affected side, stature, body mass, age, worst pain, and AKPS (Table 2). No participant was lost during follow-up, and all patients completed follow-up at six weeks. The exercise session adherence of the intervention group attending exercise program was 93%. The adherence to health education session in the intervention group and control group was 89% and 86%, respectively.

**Table 2:** Demographic data at baseline

Characteristic	Intervention group	Control group	*P* - Value
Gender (F/M)	9/10	8/11	0.75
Affected side (L/R)	11/8	9/10	0.75
Stature (m) *	1.7(0.1)	1.7(0.1)	0.91
Body mass (kg) *	67.3(9.6)	67.5(10.2)	0.95
Age (year) *	31.8(5.5)	32.3(7.0)	0.82
VAS worst pain (mm) *	50.5(12.0)	49.6(9.2)	0.79
AKPS (Score) *	74.2(9.8)	74.2(9.3)	0.99

VAS, visual analog scale; AKPS, Anterior Knee Pain Scale; F, Female; M, Male; L, left; R, right. * Data were presented as mean ± SDs

### Pain

**Fig. 2:**
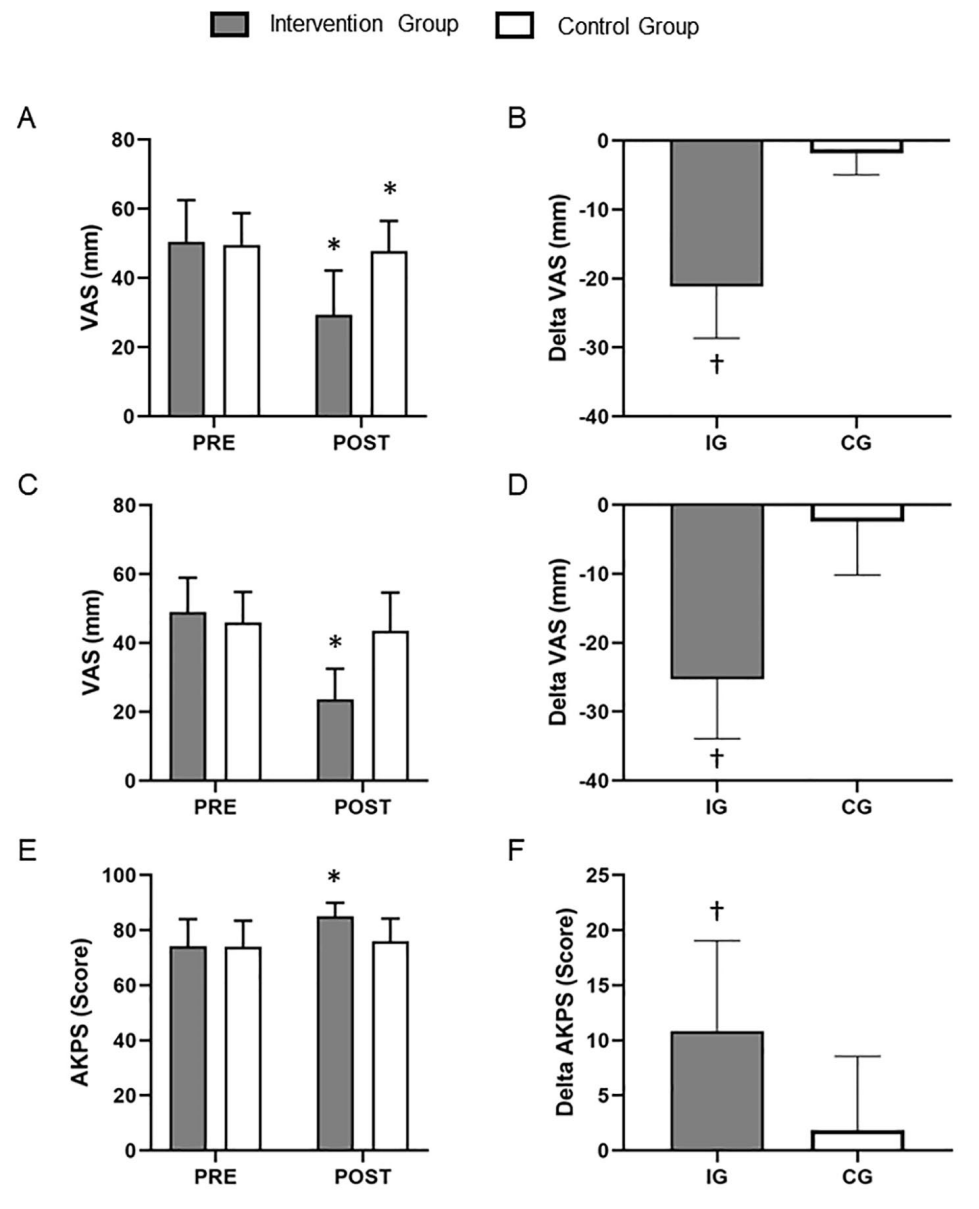
Pain and function outcomes pre- and post-intervention for patients. Panels **A**, **C** and **E** show VAS worst pain, VAS pain during ADL and Anterior Knee Pain Scores, respectively, before (PRE) and after (POST) the intervention. *P < 0.05 for within-group comparisons. Panels **B**, **D** and **F** show delta changes (PRE to POST intervention) in VAS worst pain, VAS pain during ADL and Anterior Knee Pain Scores, respectively. † *P* < 0.05 when compared with control group. IG, intervention group; CG, control group. Data are presented as mean ± SDs

No significant difference was found in pain intensity (*P* = 0.79 and 0.34 for worst pain and pain with ADL, respectively) at the baseline. At 6 weeks, within group decrease was found in the worst pain (decrease 42%, *P* < 0.01) and pain with ADL (decrease 52%, *P* < 0.01) in the intervention group. In the control group, participants had significant improvement in worst pain (decrease 4%, *P* = 0.02), but no difference in pain with ADL (decrease 5%, *P* = 0.19) between the PRE and POST intervention. Importantly, delta analysis revealed that intervention group had significantly greater increases in worst pain (P < 0.01) and pain with ADL (P < 0.01) when compared with control group (Fig. 2; Table 3).

**Table 3:** Pain, AKPS, and muscle strength of patients in Intervention group and control group

	Baseline measure		6-Week measure		0–6 Week changes		Mean difference in change between groups (95% Cl)
	IG (n = 19)	CG (n = 19)	IG (n = 19)	CG (n = 19)	IG (n = 19)	CG (n = 19)	
VAS worst pain	50.5(12.0)	49.6(9.2)	29.4(12.8)	47.8(8.8)	-21.1(7.5) *	-1.8(3.2) *	-19.3(-23.2 to -15.5) #
VAS ADL pain	49.0(10.0)	46.0(8.8)	23.7(8.8)	43.6(11.0)	-25.3(8.7) *	-2.4(7.8)	-22.9(-28.3 to -17.4) #
AKPS	74.2(9.8)	74.2(9.3)	85.1(4.9)	76.0(8.2)	10.8(8.2) *	1.8(6.7)	9.0(4.1 to 13.9) #
Knee extension	171.5(52.2)	168.0(38.6)	190.9(45.3)	167.3(37.6)	19.4(11.4) *	-0.7(3.0)	20.1(14.5 to 25.8) #
Knee flexion	108.4(36.8)	109.9(24.4)	108.9(35.9)	109.3(23.9)	0.6(6.1)	-0.6(2.4)	1.2(-1.9 to 4.3)

*A statistically significant difference in mean values compared with baseline measure (*P* < 0.01). #A statistically significant difference in mean values between intervention and control groups (P < 0.01). IG, intervention group; CG, control group; VAS, visual analog scale; AKPS, Anterior Knee Pain Scale; ADL, Activities of daily living. The measurement unit of VAS worst pain and VAS ADL pain was mm. The measurement unit of knee extension and flexion muscle strength was Nm kg^− 1^ × 100

### AKPS

There was no difference between groups in AKPS at PRE intervention (*P* = 0.99). Significant within-group increase was observed in the intervention group (increase 15%, *P* < 0.01), but not in the control group (*P* = 0.25). Delta analysis showed greater increase in the AKPS (*P* < 0.01) in the intervention group compared to control group (Fig. 2; Table 3).

### Muscle strength

The muscle strength data can be found in the Table 3. Both knee extensor strength and knee flexor strength were comparable between groups at the baseline (*P* = 0.81 and *P* = 0.88, respectively). The knee extensor strength was improved significantly (increase 11%, *P* < 0.01), whereas knee flexor strength was not significantly improved (*P* = 0.68) over six weeks in the intervention group. In the contrast, participants showed no significant improvement in knee extensor strength (*P* = 0.30) and knee flexor strength (*P* = 0.31) in control group. Additionally, the intervention group had superior improvement in knee extensor strength (*P* < 0.01), but not in knee flexor strength (*P* = 0.45) when compared with control group.

### Adverse events

No serious adverse events occurred while performing the intervention group during the trial. No patients reported using co-interventions during the study period.

## Discussion

In this randomized controlled clinical trial of patients with PFP, home-based exercise provided superior clinical outcomes to control after six weeks of follow-up. Moreover, higher knee extensor strength was observed in the intervention group than in the control group after the intervention. Therefore, we recommend home-based exercise as the basis of treatment for patients with PFP.

Exercise therapy is important, commonly used in clinical practice, and recommended by the guidelines from the Academy of Orthopaedic Physical Therapy of the American Physical Therapy Association [37]. A variety of exercises have proven effective for improving the symptoms and functions in patients with PFP, including knee and hip muscle group strengthening exercises [14, 16], hamstring stretching exercises, [38] and core/stability exercises [39]. Therefore, in the present study, our exercise program is relevant for comprehensive exercise therapy based on these types of exercise and biomechanical considerations [33].

That being said, most previous studies have focused on a supervised exercise program, and there is a lack of evidence for the effectiveness of patient self-management through exercise for PFP. One previous study found that an online exercise program without guidance from a physiotherapist is beneficial for improving pain complaints and neuromuscular performance in PFP patients [24]. In present study, the exercise protocol was provided by a physiotherapist after baseline assessment and three face-to-face guidance sessions to confirm the quality of movements during each exercise and the proper intensity and volume of exercise for patients. Despite a lack of supervision in the following exercise sessions, our results showed an improvement in pain, function, and muscular strength after six weeks of performing the exercise program. This result is supported by a previous study that showed that eight weeks of home-based exercises are equally effective at pain reduction as supervised exercises [40].

Reducing pain and improving function are the main goals of PFP rehabilitation. A previous study demonstrated that an eight-week home physical therapy program had a significant effect on pain relief and function improvement [40]. In the present study, the intervention group had a significantly greater pain reduction during daily activities than the control group, which exceeded the recommended minimum amount of difference to achieve clinical relevance [29]. Additionally, over the six weeks, a significantly greater improvement in the worst pain and anterior knee pain scores was found with intervention group compared to the control group, but these changes did not reach clinically relevance [29]. In the experimental group, patients’ pain and function significantly improved compared to baseline after the intervention, reaching the minimum clinically important difference. Similarly, Kölle et al. [24] found clinically relevant improvements in pain (27/100 points) and function (10/100 points) after the 12-week online exercise program. However, in the control group, there were no significant differences in pain and function between pre- and post-intervention, and the mean values did not reach the minimum clinically important difference. This suggests that health education alone may not be sufficient to improve patients’ pain and function. Additionally, the self-mobilization of the patella, regarded as manual therapy, also proved beneficial to PFP patients [41], but was not included in our exercise protocol. Further research is warranted to investigate whether the inclusion of self-mobilization or myofascial release exercises in the home program can result in greater improvements for patients.

Quadriceps muscle strength, which is associated with the PFP [42–44], may increase the risk of developing knee osteoarthritis [45]. Thus, restoring knee muscle strength is an important aim of PFP. According to the recommendations of the American College of Sports Medicine, a resistance load of 70–85% of an individual’s one-repetition maximum (1RM) is necessary to improve muscle hypertrophy, and a 60–70% load of 1RM is needed to develop muscular strength [46]. Quadriceps strengthening exercises for PFP are commonly prescribed at 60–70% 1RM, which is consistent with the ACSM guidelines [47, 48]. In the present study, the knee extensor improvement of the intervention group was significantly than that of the control group, but the amount of strength increase was 11%, which is relatively small. Due to the home-based protocol making it difficult for patients to realize this high training load, we used the OMNI scale to assess the adequate effort of patients, which showed submaximal knee extensor strength gains. The greater gains in quadriceps muscle strength in the intervention group is consistent with previous studies, which reported that the exercise program produces greater muscle strength gains [49]. The knee extensor improvement may be a result of both central and neural adaptation of strength training [50]. Due to our program’s exclusion of hamstring-specific training, the knee flexor improvement was not found to be significantly greater in the intervention group compared to the control group. Despite the short-term duration of the exercise protocol, strength training for more than six weeks might be adequate to identify detectable changes in muscle strength [51].

### Strengths and limitations

The major strengths of our study are that we provided a home-based exercise program for patients with guidance to make sure their proper technique during the exercise. The home-based exercise does not require any gym equipment, which makes the intervention feasible and generalizable for future implementation. Additionally, we used the regularly remote support to provide health education and adjustment of training load to create continuously adequate stimuli of muscle in the process of intervention. Other strengths that ensured the quality of the study include its use of blinded assessments and data analyses, the low participant withdrawal rate, and the high intervention adherence rate. Moreover, the outcome measures were extensive and included both clinical and isokinetic strength testing.

This study has several limitations. Due to the nature of the interventions in this study, it was not feasible to blind the participants and therapists involved in home exercise and health education. However, blinding of the assessor and statistician was carried out for the outcome measures. Hip muscle strength was not evaluated in the present study. PFP patients exhibited weaknesses in the hip abductors, which may increase stress in the patellofemoral joint. However, hip muscle strengthening was included in the exercise program due to the lack of a reliable method to evaluate the muscle strength of this muscle group in isokinetic muscle testing. The intervention program consisted of core training and stretching, in addition to quadriceps training, to provide better outcomes for patients with PFP. However, these interventions, especially stretching exercises, may also influence muscle strength. Our intervention lasted only six weeks; thus, the long-term effects of this home-based exercise program on the patients’ outcomes could not be determined. In the home-based exercise program, the intensity is largely based on individual perceived exertion. Consequently, the total physical effort of each exercise was not measured, limiting the program’s comparability to those in other studies.

## Conclusion

This study demonstrated that six weeks of home-based exercise and health education significantly improved pain and function and yielded greater knee extensor strength compared with no exercise for patients with PFP. Thus, home-based exercise and health education is indicated as a feasible option for the management of PFP. More randomized trial study with large samples is needed to confirm the long-term effects of home-based exercise on PFP in further research.

## Data Availability

The datasets used and/or analyzed during the current study will be available from the corresponding author upon reasonable request.

## Abbreviations

PFP: Patellofemoral pain
ADL: Activities of daily living
AKPS: Anterior Knee Pain Scale
VAS: Visual Analog Scale
VMO: Vastus medialis obliquus
ICCs: Intraclass correlation coefficients
1RM: One-repetition maximum
